# The Hospital Graduate Degree

**Published:** 1890-11

**Authors:** Clarence King

**Affiliations:** Machias, N. Y.


### THE HOSPITAL GRADUATE DEGREE.

*Editors Buffalo Medical and Surgical Journal:*

DURING the past few years there has arisen a popular demand, fostered by certain leading physicians and medical societies, for a radical advance in the medical qualifications of the physicians of this State. Some decided benefit has already come from the agitation of this subject, as is seen in the additional requirements now prescribed for the entrance and graduation from our medical colleges, and by the special branches taught to, and required of, the students of these schools. Nearly the whole medical fraternity unite, or appear to unite, in this demand for higher medical education. Several schemes have been advanced by different persons, some of which are of ante- and others of post-graduate application. One of the latter is the scheme advanced by Dr. Geo. E. Abbott, (*Medical Record*, April 5, 1890,) of establishing a new degree, to be known as the Hospital Graduate Degree. To the latter's indication of this degree (H. G.) he would add either M. or S., according as the work had been principally medical or surgical, and affix the name of the hospital as a further indication of the character and amount of work done. It must be evident to all that the abbreviations for such a degree, if written as the author suggests, would be bungling and inharmonious in comparison to those of the more common degrees, and would be too lengthy to employ with good taste in connection with other scientific and literary degrees of far greater value. However, Dr. Abbott advances arguments to show that the new degree would be of benefit to the public and the profession, and says "it will be one means of lifting the standard of medical education." This may be true to a limited extent, but I could not favor such a proposition as this, for the reason that it would be too exclusive, and the value of the new degree would not be great enough to compensate for it. The number of hospital internes (and consequently, as now organized, hospital graduates) is, and must be, limited in comparison to the total number of M. D.'s graduated, and hence only a few could obtain the Hospital Graduate Degree, however good their qualifications might be. I would propose the following plan, which I think is far better than Dr. Abbott's :

Our degree of Doctor of Medicine corresponds to the literary degree of Bachelor of Arts. A Bachelor of Arts, if he continues his literary studies for a certain length of time, is entitled to the degree of Master of Arts. It would be analogous if we should grant an advanced degree to those persons having an M. D. degree who could show that they had faithfully pursued their medical studies for a certain number of years since graduation, and had made material advancement in their scientific attainments, and that degree could be best called the Master of Medicine Degree. Let our post-graduate schools and those colleges that provide sufficient laboratory and clinical instruction and make provision for advanced work, grant the degree of Master of Medicine, after a personal examination of the candidate, to those graduates of a legally incorporated college who prove themselves worthy of the honor. Any work or special study accomplished by the candidate since graduation, whether it be hospital work, foreign study, or private practice, should be taken into consideration in determining his fitness for the degree. Due credit should also be given for any publication, exclusively his own, which he may present, and, if pursuing any specialty, he should have the benefit of his proficiency in that branch. It might also be well to require an attendance of a few weeks at the school from which the new degree is desired.

By this plan, it will be seen, we would have a degree that would mean something, and one which the majority of the physicians would desire. If it was thought best to attempt to increase the remuneration by law of those who fitted themselves and took the advanced degree, its possession could be made necessary to constitute eligibility to any state, county, or municipal medical office. Further, it could be made to apply to the Examiner in Lunacy, and when the State takes action in the matter of expert testimony the M. M. degree might be made requisite to an expert witness. By such means the financial inducements for higher education would be many.

By this plan the hospital interne would stand the same chance as his professional brethern, while the doctor who had spent months and money in foreign study under some celebrated teacher, would have recognition of his labor, and why should he not have "credit for work done" as well as the hospital interne ?

I am not in favor of the needless multiplication of degrees, but the great number of ignorant quacks who practise under the sanction of the State, and who have a legal claim to the degree of M. D., have brought discredit to the profession, and have reduced the high token of educational excellence nearly to the same level with the title "Prof.," which is being assumed by almost every teacher in our country schools. Justice to the educated physician seems to demand some remedy, but it should be dealt out only to the meritorious, and not as rewards to the wealthy and influential.

CLARENCE KING, M. D.

MACHIAS, N. Y., October 6, 1890.

